# The quality of avian vocal duets can be assessed independently of the spatial separation of signallers

**DOI:** 10.1038/s41598-023-43508-w

**Published:** 2023-09-30

**Authors:** Paweł Ręk, Robert D. Magrath

**Affiliations:** 1 Department of Behavioural Ecology, Faculty of Biology, Institute of Environmental Biology, Adam Mickiewicz University, Poznań, Poland; 2 Division of Ecology and Evolution, Research School of Biology, The Australian National University, Canberra, 2601 Australia

**Keywords:** Animal behaviour, Behavioural ecology, Perception

## Abstract

Interactions among groups are often mediated through signals, including coordinated calls such as duets, and the degree of temporal coordination within a group can affect signal efficacy. However, in addition to intrinsic duet quality, the spatial arrangement of callers also affects the timing of calls. So, can listeners discriminate temporal effects caused by intrinsic duet quality compared to spatial arrangement? Such discrimination would allow assessment of quality of duets produced by a pair, as distinct from transient extrinsic spatial effects. To address this issue, we studied experimentally the influence of intrinsic duet quality and spatial arrangement on the efficacy of Australian magpie-lark (*Grallina cyanoleuca*) vocal duets. Breeding pairs duet at varying distances from each other and to multiple neighbours. Coordinated duets are more effective territorial signals than uncoordinated duets, but it remains unclear whether listeners can discriminate the effects of quality and spatial arrangement. Our playback experiment showed that any deviation from perfect regularity of partners’ notes reduced duet efficacy, but that lack of coordination due to spatial separation (slower tempo and offset of notes) had a lower effect on efficacy than effects due to intrinsic quality (irregularity). Our results therefore provide experimental evidence that the temporal organisation of group vocalisations could signal coalition quality independently of spatial effects.

## Introduction

Cooperative vocal displays by pairs or groups of animals are often characterised by temporal coordination^1–6^. Coordinated signalling can play a bonding role among related individuals or in mated pairs, such as in paired siamangs (*Hylobates syndactylus*)^7^, but it can also be strategically important during interactions with other groups^8,9^. As examples, sounds allow for a numerical assessment at a distance in lions (*Panthera leo*)^10^, spotted hyenas (*Crocuta crocuta*)^11^ and subdesert mesites (*Monias benschi*)^12^; and evaluation of the spatial distribution of callers in túngara frogs (*Physalaemus pustulosus*)^13^, black-capped chickadees (*Poecile atricapillus*)^14^, eastern towhees (*Pipilo erythrophthalmus*)^15^, Australian magpie-larks (*Grallina cyanoleuca*)^16,17^ and orcas (*Orcinus orca*)^18^. The precision of timing may also allow the quality of the group or pair be assessed, as shown for duetting birds^19,20^. We focus on duets involving breeding pairs, as providing the simplest way to explore the importance of timing in assessing the quality of cooperative displays.

A listener’s assessment of duet quality is complicated by the spatial arrangement of callers and the relative position of the listener. This is because the relatively slow speed of sound means that duet production is affected by the distance callers are apart, and duet reception depends on the distance of the listener from each caller^3,16,21^. Consider an antiphonal duet, in which partners alternate notes within a duet. During production, duet tempo will decline as callers get further apart if each partner awaits the arrival of their partner's note before calling themselves^3,16,22^. Alternatively, tempo may be unaffected or less affected if partners use visual components of display to time contributions^16^. Duet reception is also affected by the relative distance to each caller because notes from the more distant caller will arrive after a delay, so the contributions of different callers are offset^16,23^. In extreme cases, when the difference in distance is large, the offset may cause notes to overlap. Information on spatial arrangement of callers is useful in indicating readiness for immediate, joint cooperation^17,22,23^. However, given that the spatial arrangement of callers and listeners is not an intrinsic property of duet quality, listeners may prioritise temporal features of duets that are under immediate control by callers, and so more likely related to coalition quality. Here we consider intrinsic duet “quality” to include both the skill of individuals, which can be related to their experience together^19^, and their motivation, as both of these properties of the pair are likely to affect territorial defence.

**Figure 1:**
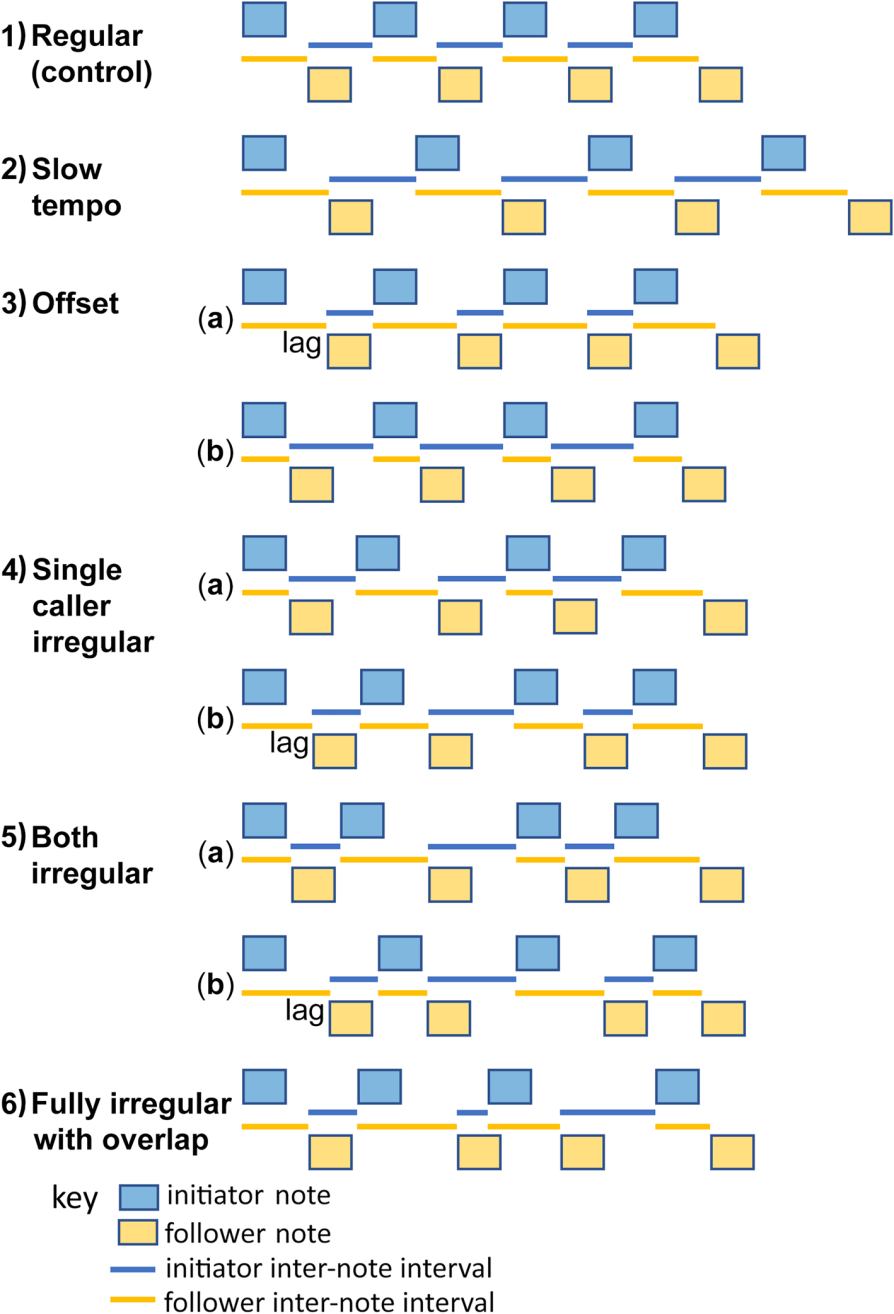
The temporal arrangement of song notes in treatments. See Table 1 for further description of treatments.

The quality of cooperative signallers is likely to affect the precision of responses to their partner, independently of any spatial effects on tempo and offset. This is important, because listeners should be selected to discriminate signals of coalition quality from the temporary cues of spatial arrangement of callers, yet there has been no experimental discrimination of these possibilities. Nevertheless, this task is difficult as, even in the production phase, irregularities can arise in many ways (Fig. 1, Table 1). The duet is formed when the duet receiver answers the initiator’s song. Therefore, when the initiator sings independently, the regularity of a duet will be determined primarily by responder’s calling. Robin chats (*Cossypha heuglini*), for example, sing several categories of duets. In one of them, the male’s singing is independent of the female’s response. Coordination is then relatively low and the female’s singing appears to be directed towards interaction with the mate, since pairs do not signal this way when interacting with others^24^. By contrast, when the initiator and the responder react to each other note-by-note, the quality of the duet will be the result of both partners’ quality. Robin chats call in note-by-note alternation in their interactions with other pairs^24^, suggesting that this type of mutual coordination does signal group cohesion. Apart from the influence on timing, the lack of regularity of partners may, in extreme cases, lead to overlapping of the partners’ notes. Where they are usually produced alternately, overlapping may therefore signal inexperienced partners and not just spatial separation^16,19^.

**Table 1:** Description of treatments. The visualizations are shown in Fig. 1.

Main treatments	Treatment details	Regularity of inter-note intervals
**(A) Control**		
1. Regular, normal tempo	Normal tempo of callers together	Both have similar, constant inter-note intervals, at normal tempo
**(B) Effects of spatial location of callers and listener: each bird has constant inter-note intervals, but may differ from each other**		
2. Regular, slow tempo	Duetters apart with slow tempo, listener equidistant	Both have similar, constant inter-note intervals, at slow tempo
3. Regular, with offset	a. Duetters apart, responder lag (listener closer to initiator)	Each has constant inter-note intervals, but different from each other
	b. Duetters apart, no responder lag (listener closer to responder)	
**(C) Effects of intrinsic quality: one or both birds have irregular inter-note intervals**		
4. Single caller irregular	a. Follower has irregular inter-note interval, no responder lag	Partial irregularity; one has constant inter-note intervals, other irregular
	b. Initiator has irregular inter-note interval, responder lag	
5. Both irregular, copying, no responder lag	a. Both have irregular inter-note intervals, no responder lag	Copied irregularity; one copies the other's irregular inter-note intervals
	b. Both have irregular inter-note intervals, responder lag	
6. Fully irregular, no copying, overlap	Both have irregular inter-note intervals, including note overlap	Full irregularity with overlap; neither is coordinated with the other

We tested the effect of temporal organisation on efficacy of vocal duets in the Australian magpie-lark. Pairs sing precise antiphonal duets in defence of the territory, and coordinated duets elicit stronger responses than uncoordinated ones^19^. Furthermore, the level of coordination increases with the pair’s tenure, so that coordination is likely to signal skill or motivation for defence^19^. However, in previous experimental work, coordinated and uncoordinated playbacks differed in multiple ways, so it was not possible to tell what features of temporal organisation affected response. In subsequent studies, birds responded less to offset duets than to regular ones, but the experiments were designed to examine audio-visual communication, so that the treatments also included robotic taxidermic models^16,17^. Here, we test experimentally the impact of temporal coordination of vocal duets related to both spatial separation and intrinsic duet quality. We distinguished six categories of temporal distribution of notes within a duet (Fig. 1). We predicted that subjects would respond most strongly to regular duets with a normal tempo, at intermediate levels to duets with cues of spatial separation of callers, and least to irregular duets that indicate reduced coalition quality. The tempo of duets naturally decreases as partners separate, and separation can result in an offset if the receiver is at different distances from both senders^3,16^. Similarly, as magpie-larks coordinate their songs note-by-note, the loss of regularity in a pair can occur due to actions of one or both partners, which in extreme cases can result in overlapping notes^3,19^. Therefore, the treatments we performed reflect the effects that arise naturally.

## Results

**Figure 2:**
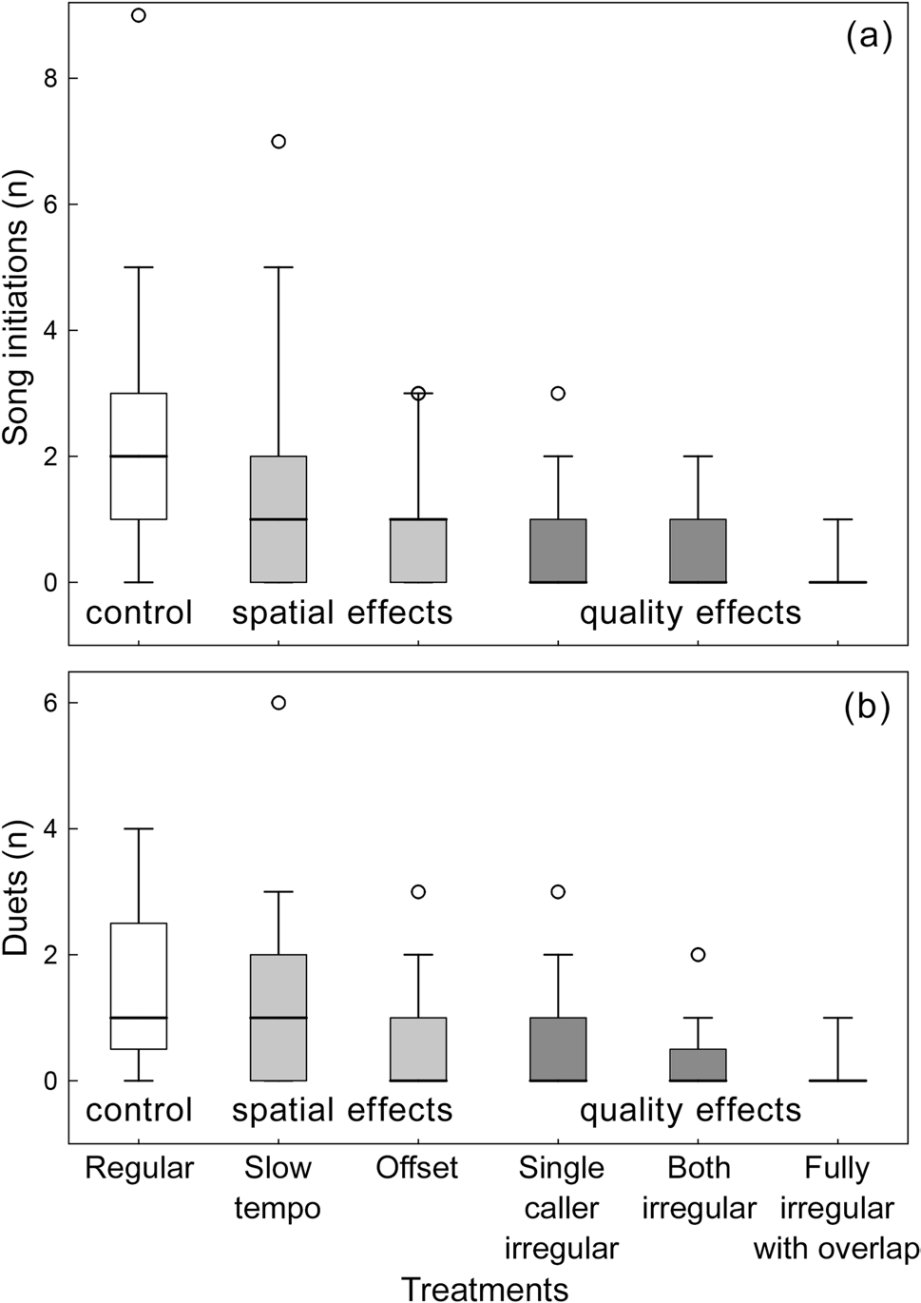
The boxplot of the number of songs initiated by individuals (**a**) and duets produced by pairs (**b**) during treatments. Boxes show medians (thick lines) and interquartile ranges (Q_1_–Q_3_), whiskers show 5th and 95th percentiles, and circles show outliers. Statistical analyses are in the text and Tables 2 and S1.

As predicted, birds were most responsive to regular, fast duets, and least responsive to temporal variation associated with low duet quality rather than spatial separation. Overall, there was a strong effect of playback treatment on both measures of response (number of song initiations: Wald *Χ*^2^_5_ = 110.31, *P* < 0.001; number of duets: Wald *Χ*^2^_5_ = 83.20, *P* < 0.001), and birds responded more to duets associated with spatial effects than to quality effects (Fig. 2a,b; song initiations: B ± SE = 0.37 ± 0.08, 95% CI 0.22–0.52; Wald *Χ*^2^_1_ = 22.48, *P* < 0.001; number of duets: B ± SE = 0.36 ± 0.06, 95% CI 0.23–0.49; Wald *Χ*^2^_1_ = 30.99, *P* < 0.001; Table S1). Furthermore, birds initiated the most songs and performed duets in response to the regular, fast treatment (Fig. 2a,b; all pairwise comparisons *P* < 0.006; Table 2), more songs and duets to the slow tempo than to offset and irregular treatments (Fig. 2a,b; all *P* < 0.014; Table 2), and more songs to offset than to irregular treatments (Fig. 2a,b; all *P* < 0.027; Table 2). Males initiated more songs than females (males: 0.98 ± 0.09, females: 0.38 ± 0.06; Wald *Χ*^2^_1_ = 31.97, *P* < 0.001; Table S1), but this difference was similar among treatments (interaction between sex and treatment: Wald *Χ*^2^_6_ = 7.02, *P* = 0.219).

**Table 2:** Pairwise comparisons between treatment means (Fisher's LSD test).

Treatments compared *(i*) vs (*j*)		Song initiations				Duets			
		Mean	SD	95% CI	*P*	Mean	SD	95% CI	*P*
Regular (1)	(2)	2.21	1.77	1.69 – 2.72	**0.006**	1.63	1.42	1.21 – 2.04	**0.008**
	(3)				** < 0.001**				** < 0.001**
	(4)				** < 0.001**				** < 0.001**
	(5)				** < 0.001**				** < 0.001**
	(6)				** < 0.001**				** < 0.001**
Slow tempo (2)	(3)	1.46	1.56	1.01 – 1.91	**0.014**	1.15	1.27	0.78 – 1.52	** < 0.001**
	(4)				** < 0.001**				** < 0.001**
	(5)				** < 0.001**				** < 0.001**
	(6)				** < 0.001**				** < 0.001**
Offset (3)	(4)	0.90	1.13	0.57 – 1.23	**0.027**	0.48	0.74	0.26 – 0.70	0.365
	(5)				**0.007**				0.090
	(6)				** < 0.001**				**0.001**
Single caller									
irregular (4)	(5)	0.50	0.77	0.28 – 0.72	0.467	0.46	0.74	0.24 – 0.67	0.389
	(6)				**0.022**				**0.009**
Both irregular (5)	(6)	0.42	1.13	0.57 – 1.23	0.146	0.29	0.54	0.13 – 0.45	0.086
Fully irregular									
with overlap (6)		0.19	0.39	0.07 – 0.30		0.08	0.28	0.00 – 0.16	

## Discussion

Our experiment confirms that magpie larks distinguish short-term temporal variability of duets resulting from spatial effects from that resulting from intrinsic duet quality. Birds responded more weakly when there was any deviation from perfect regularity, which confirms that duet timing is crucial for them in territorial interactions. At the same time, however, the temporal effects of spatial separation (slower tempo and offset of notes) had less impact on response than the effects of intrinsic duet quality (irregularity). Our results therefore provide experimental evidence that duet timing could signal the skill or motivation of the coalition independently of spatial cues.

Spatial effects on duet tempo and timing did affect responses to playbacks, potentially because the current spatial separation of callers is relevant to territorial defence. Consistent with our previous results^16,17^, there was a weakened response to duets with slow tempo or offset, which are both consequences spatial separation. The treatments in previous experiments, however, combined the vocal duet with visual display movements of taxidermic models. These visual signals influenced the reactions to songs yet, unlike acoustic signals, visual ones are not affected by temporal delays. Therefore, the results of those experiments could not isolate the impact on response of spatial effects on signal timing. By contrast, the current experiment isolated the acoustic component of the duet display, which is subject to spatial effects because of the slow speed of sound. We suggest that reduction in response to slow and offset duets is precisely because these features betray the physical separation of signallers, which indicates that the pair is not currently united. This type of tactical information can be important during territorial interactions as it allows for the right defensive strategy. As an example, wolves that live in larger packs respond intensely to vocalisations of strangers when together and weakly when dispersed. At the same time, the dispersal of the group usually occurs after the end of the common howling session^25^. These observations suggest that group members signal cohesion but conceal dispersion.

Although tempo and offset are both outcomes of spatial separation, tempo had less effect than offset, suggesting that other factors are also important. We suggest that this is because tempo is a poorer cue of spatial separation, for two reasons. First, tempo is a less reliable indication of separation because partners can sing at different tempos for a given spatial separation^16^. Magpie-larks have several types of song notes within their repertoire, differing in length, and this choice alone can affect the duet tempo. However, partners may also choose a specific tempo for a given duet type based on shared experience and learning^3^. Second, tempo does not fall as rapidly with distance as expected from the speed of sound, probably because birds partly use visual cues to time responses to their partner’s songs^16^. In contrast to tempo, offset is unaffected by tempo and is an inevitable consequence of spatial separation when the listener is closer to one of the callers. Furthermore, unlike the change of tempo with partners’ separation, which occurs in both acoustic and visual components of the duet display, offset is a purely acoustic phenomenon. Therefore, the visual component is not relevant to interpret offset.

As predicted, temporal cues of intrinsic duet quality had a greater effect on response to duets than cues of spatial separation. Birds reacted most strongly to duets that were fully regular and with a fast tempo, less to duets with slow tempo or offset, which indicate spatial separation, and least to irregular duets, which are likely to indicate less experienced pairs (although in pairwise comparisons offset was significant only for the number of songs, not duets). Furthermore, the weakest reaction was caused by completely irregular duets. Such poorly coordinated duets are produced mainly by less experienced pairs^19^. These poorly coordinated duets also included overlapping of notes, and it is possible that overlapping is itself an additional cue of quality, independent of irregularity, but this is unknown^19^. Overlapping of notes may make the timing of notes difficult to assess, reducing the ability to gain information from temporal cues. Furthermore, overlapping might indicate conflict within the signalling pair. Many songbirds avoid acoustic interference by short-term adjustment of song timing^26,27^, which suggests that overlapping could be related to competition rather than cooperation. Consistent with this, duetting antbird females respond to unpaired females by overlapping the signals of their own mates, who in turn compensated by adjusting their signals to avoid interference^28^. Regardless of the specific cues used or presence of conflict, being able to assess intrinsic duet quality, as distinct from spatial effects, is likely to be of benefit when defending the territory, such as by prioritising defence against pairs that represent greater threats.

Many previous studies have pointed to the negative consequences of lack of coordination, but have not discriminated effects due to spatial separation compared to intrinsic effects of skill or motivation. An earlier experiment on magpie-larks found that coordinated duets elicit stronger responses than uncoordinated ones, but the uncoordinated treatment differed in a variety of temporal parameters, so it was unclear which specific features affected response^19^. A similar effect of coordination was found for duetting rufous-and-white wrens (*Thryophilus rufalbus*) that responded more intensely to coordinated than uncoordinated duets, but again specific cues were not resolved^5^.

Group signals such as vocal duets and choruses are very common in animals, and separation of senders is inherent in this form of signalling^8,29^. Spatial effects are therefore a component of signalling for many species. In amphibians and insects that call in choruses, females tend to approach the leading sound^30^. This type of preference is straightforward when the leading call actually precedes others^31^, however, reverberations can distort this pattern and lead to perceptual fusion and moving towards phantom or reflected positions^32,33^. Because the calls of frogs and insects are pulsating in structure, precedence will inevitably depend on the receiver's position relative to the senders. However, it can also arise as a result of deliberate masking and anticipation of competitor's calls^31^. Such intentional interference will be effective, given that precedence works independently of the amplitude of the calls^31^, and thus of the distance cue, and seems to be the effect of perceptual bias^30^. However, there are no studies on precedence that would unambiguously separate the influence of interactions between calling males from their actual spatial distribution.

In addition to acoustic signals, spatial effects on assessment of signals arise in other modalities. Visual signals are independent of spatial effects entailing time delays, but spatial arrangement still plays a role in visual signalling and perception. For example, male bowerbirds create courts with grey and white objects that increase in size with the distance from the entrance to the bower. This gradient creates a forced visual perspective for the female, which can yield false perception of size and distance^34^. Chemical signals are subject to even greater spatial effects, although it is not at all certain whether timing has any significance in this form of communication, taking into account the characteristics of this medium. Chemical diffusion, which is the main mechanism of chemical propagation, is non-directional and slow because, instead of waves, it requires the movement of molecules that dilute rapidly in a medium that is also moving. As a result, all temporal patterns in chemical propagation are lost at a small distance from the source^35^, and little is known about the role of spatial effects in chemical communication, especially considering the vast spectrum of substances that may be involved in signalling. Spatial effects can also play a role in the assessment substrate-borne vibrational signals of invertebrates^36^. Because different parts of the plant have different and specific frequencies at which vibrational energy propagates particularly well, the type of substrate may have a potential influence on the distance estimation to the sender^37^. Overall, there are many opportunities to study the effects of spatial separation on communication.

## Materials and methods

### Study site and species

We studied a population of magpie-larks in parks and suburbs of Canberra, Australia. Magpie-larks inhabit open woodland, and suburban parks and gardens, through most of Australia^38,39^. Pairs defend territories throughout the year^40^, and the sexes are easily distinguishable by plumage differences^41^. Males and females each produce solo songs, and can combine songs to form duets. Duets are typically antiphonal and can be initiated by either sex. Solo songs consist of a series of short notes (these are actually ‘motifs’, including one or more elements^3^), which are 300–600 ms long and given at a tempo of about 1 note/s. Individuals have a repertoire of about three to six different song types, each composed of a single, repeated note^3^. In duets, partners alternate their notes on average six or seven times. Each bird uses the same note type throughout a duet, but it is rare for pairs to sing a duet in which each bird uses the same note type^3,42^. The timing of partners’ notes is often precise, with perfect alternation and a regular tempo of about 1 note/bird per second. Pairs that have been together longer are more likely to produce more coordinated duets, which are more threatening territorial signals^19^. Furthermore, studies using taxidermic models have shown that vocalizations indicate the number of individuals and their spatial distribution^16,17,43^.

### Experimental design and treatments

We broadcast magpie-lark duets to test the effect of temporal variation cues indicating spatial separation or intrinsic duet quality. We used purely acoustic playbacks, with a single speaker, as opposed to our earlier audio-visual studies with two speakers^16,17,44^, to test specifically for the effects of acoustic timing, independently of their interactions with visual cues and direct spatial effects. We broadcast six treatments, in randomised order, on 144 mated pairs of magpie-larks, with each treatment carried out on 24 pairs. The creation of treatments was similar; they differed only in the temporal distribution of male and female notes (Fig. 1, Fig. S1, Table 1), to reflect both spatial and quality effects on coordination, as explained below.

1. Regular, normal tempo (control): male and female notes were alternated with perfect regularity of inter-note intervals, defined as the period from the start of one note to the start of the previous note by the other bird, and produced with even tempos of 1 note/s for each bird (Fig. 1.1, Table 1). Such a tempo corresponds to the natural tempo of a duet for birds next to each other^16^.
2. Regular, slow tempo: male and female notes were alternated with perfect regularity and even tempos, but compared with the control each note was delayed by 59 ms. This delay corresponds to a separation of 20 m between the male and female assuming the speed of sound is 340 m/s and birds await the arrival of their partner’s note (Fig. 1.2, Table 1). Magpie-larks duet at a slower tempo when separated from each other^3,16^.
3. Regular with offset: the contributions of the two callers were offset by 59 ms, as if one caller was 20 m further away than another relative to the receiver (again assuming sound travelled at 340 m/s). From the perspective of the receiver, when the duet is offset the notes of the further sender are delayed in relation to the closer one. As a result, the inter-note intervals of one individual are long and of the other is short, so that a whole duet sounds as if the consecutive intervals were alternately longer and shorter. To isolate the effect of offset from the slower tempo of separated partners, we did not add delays between the notes of the partners. This timing corresponds to a situation in which partners respond to each other based on visual cues^16^. Depending on whether the duet is initiated by the nearer or further sender, the first interval is either longer or shorter than the second. Therefore, half of birds (12) received a treatment variant with a duet initiated by the closer bird and half by the further bird (Fig. 1.3, Table 1).
4. Single caller irregular: the contributions of one caller were played back with regular inter-note intervals, while the other bird’s notes were played back alternately with short and long intervals. This pattern cannot arise merely from spatial separation, and therefore is related to intrinsic duet quality. In this treatment, half of birds (12) received a duet in which the duet initiator called with regular inter-note intervals and half in which the responder called regularly (Fig. 1.4, Table 1).
5. Both callers irregular: the contributions of duetters were played back with alternately long and short or short and long inter-note intervals. It is characterized by an even tempo of the bird that copies. This pattern also cannot arise merely from spatial separation, but could arise if one bird copied its partner's irregular inter-note intervals. Again, half of birds (12) received a duet without lag, that is when the responder was the copier, and half with the lag, that is when the initiator was the copier (Fig. 1.5, Table 1).
6. Fully irregular with overlap: each partner sings at variable and different tempo, not copying their partner, a consequence of which was the occasional overlapping of partners' notes (Fig. 1.6, Table 1). This represented the least coordinated treatment, indicating the lowest duet quality. Each playback was different, but all were similar in total duration (± 50 ms) to the control duet. To achieve this, we drew positive and negative lengths of intervals from the normal distribution, but only allowed the overlap of two notes in a playback.

**Figure 3:**
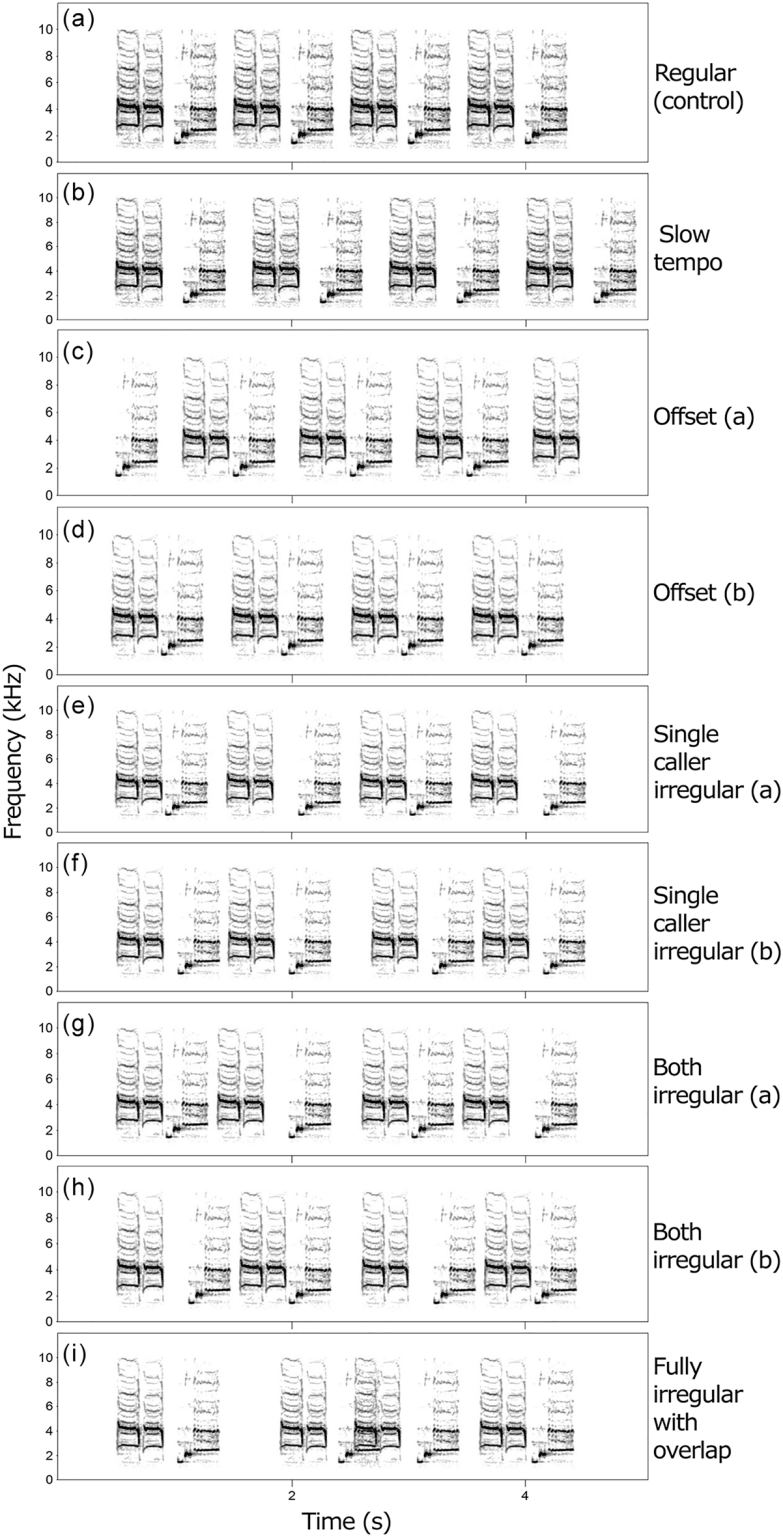
Sonograms of playbacks used in treatments: (**a**) regular (control) duet, (**b**) slow tempo duet, (**c**) duet with offset and responder lag, (**d**) duet with offset and without responder lag, (**e**) single caller irregular duet without responder lag, (**f**) single caller irregular duet with responder lag, (**g**) both callers irregular duet without responder lag, (**h**) both callers irregular duet with responder lag, (**i**) fully irregular duet with overlap. All song types can be produced and initiated by both sexes. See Table 1 for further description of treatments.

Playbacks originated from high-quality recordings of duets from 144 local pairs, so that each pair received a unique exemplar from unfamiliar birds (> 2 km). Recordings were from an Olympus LS-11 and Sennheiser ME66 microphone, sampling wave files at 48 kHz and 16 bits. The files were high-pass filtered individually to remove sound below the notes, based on visual inspection, and the male and female tracks were composed using Avisoft-SASLab Pro software (Avisoft Bioacoustics, Berlin, Germany). Every duet playback contained eight notes that were formed by pasting male and female notes into a stereo wave file in alternation, and lasted about 4 s for control playbacks to 4.5 s for slow tempo playbacks, which is natural timing (Fig. 3). To further standardize the timing of notes in treatments, we used only the two most common types of notes in each playback, which are sung by all pairs and regardless of sex (motifs 1 and 3 as described in^3^). In each treatment, half of the duets started with each sex and half started with each kind of note.

### Playback field methods

Playbacks were carried out in October and November 2019, between 0630 and 1115 h on days without rain or strong wind. Playbacks were initiated only if partners were together and within 25 m of the speaker, and if they had not engaged in any interaction with neighbours or sung during the minute before each treatment. In our earlier study, the birds sang only once on average for five minutes before playback^42^, so a one-minute pause is therefore common. Each pair of birds received only one playback. Playbacks were broadcast from a JBL Xtreme amplified stereo loudspeaker (40W, frequency range 70–20,000 Hz). Songs were broadcast at natural amplitudes of 66–70 dB SPL at 10 m, measured by a RadioShack Sound Level Meter see also^38,42,45^.

We measured the response to playback in the 1 min period starting from the onset of the playback. We counted the number of songs initiated by males and females, including solos and duets initiated by each individual, and the number of duets produced by a pair. Solo songs and duet initiations represent individual-based behaviours that are a proxy of territorial response of individuals, whereas duets require a response to the partner’s song, and thus they represent a proxy of cooperative territorial defence. Song rate, particularly of males, is the most sensitive measure of response to territorial threat in magpie-larks^42,45^. Therefore, we expect males to initiate more songs than females over all treatments^42^. Given the low background rate of song^42^ (above), song rate in the 1 min sample period is a good measure of the reaction to playback.

### Statistical analysis

To compare the effects of experimental treatments on responses of birds we used generalized linear models (GENLIN) and their extension generalized estimating equations (GEE). Both procedures are suitable for non-normally distributed data and both allow the use of heteroskedasticity-consistent standard errors^46^. Unlike mixed models, which estimate subject-specific effects (random effects), GENLIN and GEE estimate population-averaged effects and are therefore appropriate for ecology or social sciences. We carried out separate analyses for each response variable. To compare the number of songs initiated by paired males and females we used GEE, which is appropriate for paired measurements, whereas to compare the number of duets by pairs we used GENLIN. Within GENLIN and GEE, we used an a priori contrast of the responses to “spatial effects” and “quality effects” (Fig. 2) and post-hoc Fisher's LSD method to create confidence intervals and compare means of all treatments. We fitted the variables using log-normal distribution^47^. In the analysis of song initiations, we present the statistics of the main effects: sex and treatment, after the nonsignificant interaction effect was dropped. This selection was based on the lower information criteria of the model without interaction (QIC_u_ 314.62 vs. 333.33)^48^. The analyses used SPSS 28 software. All P-values were two-tailed, and means ± SE are given.

### Ethics statement

All experimental protocols were approved by the Australian National University Ethics Committee (A2018/10), Australian Bird and Bat Banding Scheme, and Environment ACT. The use of the animals adhered to guidelines for animal research in Australia (Health and Medical Research Council 2013).

### Supplementary Information

Supplementary Information 1.

Supplementary Table 1.

## Data Availability

The data reported in this paper are available in electronic supplementary material, Dataset S1.
