# Supplementary material for: The quality of avian vocal duets can be assessed independently of the spatial separation of signallers
Source: Sci Rep. 2023 Sep 30;13:16438. doi: 10.1038/s41598-023-43508-w (PMC10543378; doi:10.1038/s41598-023-43508-w)
Supplement: Supplementary file 2 — Supplementary Table 1. [file 41598_2023_43508_MOESM2_ESM.pdf]

Table S1. Estimates of parameters in models of vocal reaction of individuals and pairs to treatments.

Dependent		Predictor	B	SE	95% CI		Wald X^2_1	P
Song initiations [1]		Intercept	-1.27	0.32	-1.91	-0.63	15.31	< 0.001
	Sex	Female	-0.93	0.16	-1.25	-0.61	31.97	< 0.001
		Male	0					
	Treatment	Regular	2.42	0.34	1.76	3.09	50.51	< 0.001
		Slow	2.02	0.34	1.36	2.68	35.91	< 0.001
		Offset	1.54	0.38	0.80	2.28	16.75	< 0.001
		Single irregular	0.87	0.39	0.11	1.63	5.05	0.025
		Both irregular	0.63	0.42	-0.20	1.45	2.22	0.136
		Overlap	0					
Duets [2]		Intercept	-2.08	0.54	-3.14	-1.02	14.83	< 0.001
	Treatment	Regular	3.00	0.55	1.92	4.08	29.59	< 0.001
		Slow	2.57	0.55	1.48	3.65	21.51	< 0.001
		Offset	1.79	0.59	0.63	2.95	9.21	0.002
		Single irregular	1.47	0.60	0.29	2.65	5.93	0.015
		Both irregular	1.10	0.64	-0.16	2.35	2.94	0.086
		Overlap	0					

(1) GEE, (2) GENLIN. In both models the fully irregular treatment with 'overlap' was set as a reference category.
